# Dust flow analysis by low coherence Doppler lidar

**DOI:** 10.1038/s41598-023-30346-z

**Published:** 2023-03-11

**Authors:** Kosuke Okubo, Nofel Lagrosas, Tatsuo Shiina

**Affiliations:** Graduate School of Science and Engineering, Chiba University, Chiba-Shi, Chiba 263-8522 Japan

**Keywords:** Environmental sciences, Engineering, Optics and photonics

## Abstract

Visualization of dust flow and wind dynamics near the ground surface are essential for understanding the mixing and interaction between geosphere and atmosphere near the surface. Knowing the temporal dust flow is beneficial in dealing with air pollution and health issues. Dust flows near the ground surface are difficult to monitor because of their small temporal and spatial scale. In this study, we propose a low-coherence Doppler lidar (LCDL) for measuring dust flow near the ground with high temporal and spatial resolutions of 5 ms and 1 m, respectively. We demonstrate the performance of LCDL in laboratory experiments using flour and calcium carbonate particles released into the wind tunnel. LCDL experiment results show a good agreement with anemometer measurement in wind speeds ranging from 0 to 5 m/s. The LCDL technique can reveal dust’s speed distribution, which is affected by mass and particle size. As a result, different speed distribution profiles can be used to determine dust type. The simulation results of dust flow coincide well with the experimental results.

## Introduction

Dust flows are active near the ground surface, where wind flow is complex. It is important for understanding the mixing and the interaction between the geology and the surface atmosphere. The scattering of dust deposited on the ground surface is a major problem not only for environmental conservation, but also for human health such as respiratory diseases, and for air pollution by anthropogenic dust in urban areas^1–3^. In particular, the dust flow in the lower atmosphere is complicated by topography and structures. The behavior of the scattering dust in the field is steep. By visualizing the local urban winds among buildings, called street canyons^4^, distribution of dust flow in local area can be predicted, and its impact understood on living areas. Near the surface atmosphere, certain obstacles such as mountains and buildings block and sharply change the dust flow. The upper atmosphere, on the other hand, has few obstacles and dust flow is somewhat gradual. The wind flow in the atmosphere depends on altitude^5^. The higher altitude is, the larger the mass of air cell is, and the vertical upper atmosphere has larger spatial and temporal scale^6^. The strong demand for wind flow measurements has been in the vertical upper atmosphere from the standpoint of safety for aircraft taking off and landing and efficiently control of wind-power plant^7,8^. Propeller-type anemometers, radiosondes, Doppler sodars, and Doppler lidars are used for wind measurements^9–12^. In-situ anemometers require its installation in the measurement space, while it may change the wind field itself. Doppler sodar and Doppler lidar, on the other hand, can remotely acquire the wind information in the measurement^13,14^. They are effective for long-distance wind field measurements^15^. Doppler lidar is installed into airports and measure the vertical upper atmosphere over a wide measurement range from 200 m to several kilometers for a long period of time of several minutes in accordance with the large spatial and temporal scale of the atmosphere^16–18^. The nacelle-mounted Doppler lidar is installed into wind power plants and measures the horizontal atmosphere^19^. The spatial resolution of the measurement is still as large as several tens of meters. Conventional dust samplers collect dust for a certain period of time. However, this method cannot produce real-time information on dust transport. Remote sensing is the best option to detect dust near the ground since the wind field is not disturbed during the measurement^20,21^. High-resolution and high-speed measurements are essential criteria for detecting dust and aerosols near the ground. The current conventional Doppler lidar cannot capture the localized and constantly changing dust flow near the ground since the lower atmosphere has a small spatial and temporal scale of a few seconds and meters. Particle tracking velocimetry using sheet lasers is also available, but does not provide quantitative results. It has significant limitations, such as the need for dark surroundings, and is not convenient for field use^22–24^. In this paper, we develop a horizontally pointing low coherence Doppler lidar (LCDL) with high spatial and temporal resolutions of 1 m and 5 ms, respectively, to measure local dust flow. LCDL is a kind of low-coherence optical interferometer. An interference signal is obtained only when the difference of optical path length between the reference and measurement paths matches the coherence length. In addition, to monitor sudden changes in dust flow, the integration time is shortened to milliseconds for high-speed measurement. This paper’s objectives are: (1) to design and develop the concept LCDL system, (2) to verify the performance of the LDCL system, and (3) to apply this method to different scatterers and evaluate the scatters’ speed distribution.

## Low coherence Doppler lidar

### Concept

**Figure 1:**
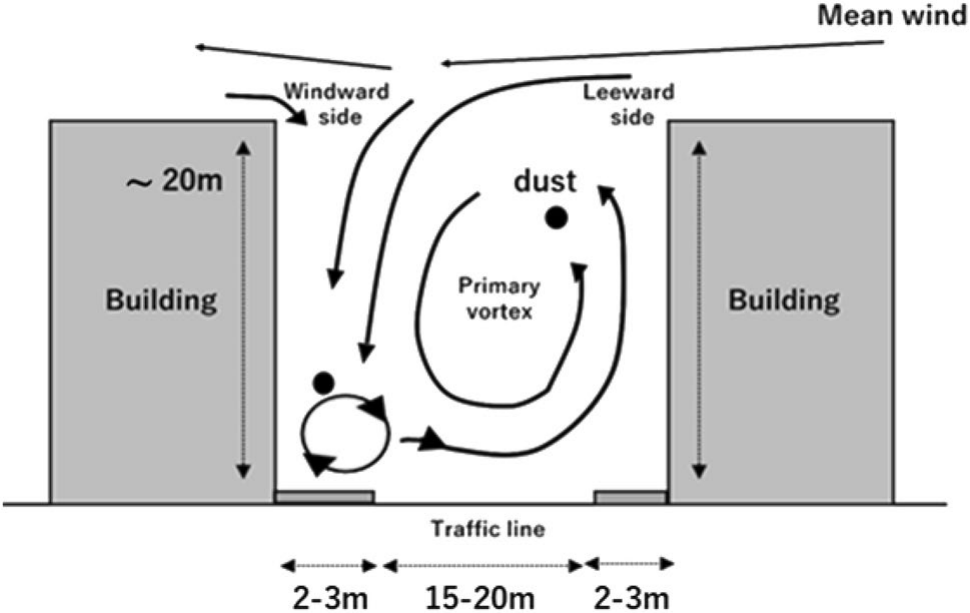
Mixing and interaction between the atmosphere and the ground surface. The wind fields generated by buildings and asphalts show how dust flows on a small spatio-temporal scale^4^.

The size of the wind field depends on the altitude. Air cells at lower altitudes are smaller and their movement is faster. The lower atmosphere, like the wind field in street canyon, is strongly influenced by the structure and ground texture. Figure 1 shows the schematic diagram of the wind field in the lower atmosphere. At an altitude range of a few meters above the ground surface, the size of the wind field is on the order of one to several meters, and the turbulence is on the order of a few seconds. In addition, monitoring the detailed dust flow at low concentrations requires optically sensitive sensors. Because the spatio-temporal scale of the lower atmosphere is small, the mixing of suspended dust in the atmosphere is rapid, and its behavior is complex^25^. In urban areas, strong winds result from heat convection due to concrete buildings and asphalt roads^26,27^. In these areas, there are hot spots of air pollutants due to the mixing of soil and air^28^. Visualization of these scatterers in the lower atmosphere accelerates the understanding of dust flow that can aid in mitigating air pollution and health problems. The contribution of the surface atmosphere to sea waves can also be studied by monitoring the interaction between the sea surface and the atmosphere. Conventional Doppler lidars measure large wind fields on its long spatial and temporal scale (hundreds of meters and minutes) above the ground. In contrast, the LCDL horizontally measures suspended dust within a small wind field on its small spatial and temporal scale (a few meters and seconds) near the ground surface.

We propose a low coherence Doppler lidar (LCDL) that is based on a low coherence optical interferometer^29,30^. Doppler lidars can be classified into continuous wave laser and pulsed wave laser use^13^. Conventional Doppler lidars include Zephyr, Wind cube, and Leosphere^31^. In recent years, Doppler lidars employing pulsed wave lasers are commonly used^32^. In our system, LCDL uses a continuous wave laser. Doppler lidar detection methods can also be classified into optical homodyne^33^ and heterodyne detections^34–36^. The difference between homodyne and heterodyne detection is found in the signal and local oscillator frequency. In homodyne detection, the signal and local oscillator frequency are equal. This feature is not the case for heterodyne detection. Optical homodyne detection is adopted for LCDL because it generally has a higher receiving sensitivity than optical heterodyne detection. Most Doppler lidars have optical heterodyne detection^37^. The LCDL system is a homodyne system, which does not require an Acousto-Optic Modulator (AOM) and a local oscillator because the light source is a continuous wave and the lidar echo interferes with its reference lights. This simplifies the optical design. The low-coherence light source allows measurements of a high spatial resolution. In order to match the small wind field in the lower atmosphere, the spectral width of the low-coherence light source is set to a few picometers resulting in a spectrum producing a low coherent length and higher spatial resolution of less than 1 m. In addition, the sampling time is shortened to milliseconds for high-speed measurement to capture the rapid flow of suspended dust. The sampling time corresponds to the time resolution. Compared to conventional Doppler lidars, which have a coherent length of several 100 m and a temporal resolution of a few minutes, LCDL has a higher resolution in both space and time. Thus, the LCDL system obtains dust speed from the Doppler-shifted interference signal frequency by employing fast Fourier transform (FFT) analysis. In the analysis of the data, the frequency resolution corresponds to the length of the FFT frame. The lidar measures the Doppler shifted frequency signal from the air/dust to determine its line-of-sight velocity, *V*, and is measured as^38^

1 $$V=\frac{1}{2} {f}_{d}{\lambda }_{0}$$

where f_d_ is the Doppler shift frequency, and λ_0_ is the light source center wavelength.

The transmitted light is partially used as reference light. The received light from the telescope is combined with the reference light in a fiber coupler to obtain interference signals. In this process, the reference path length determines the measurement distance. The distance can be freely changed  by adjusting  the reference path fiber length.

### Design

**Figure 2:**
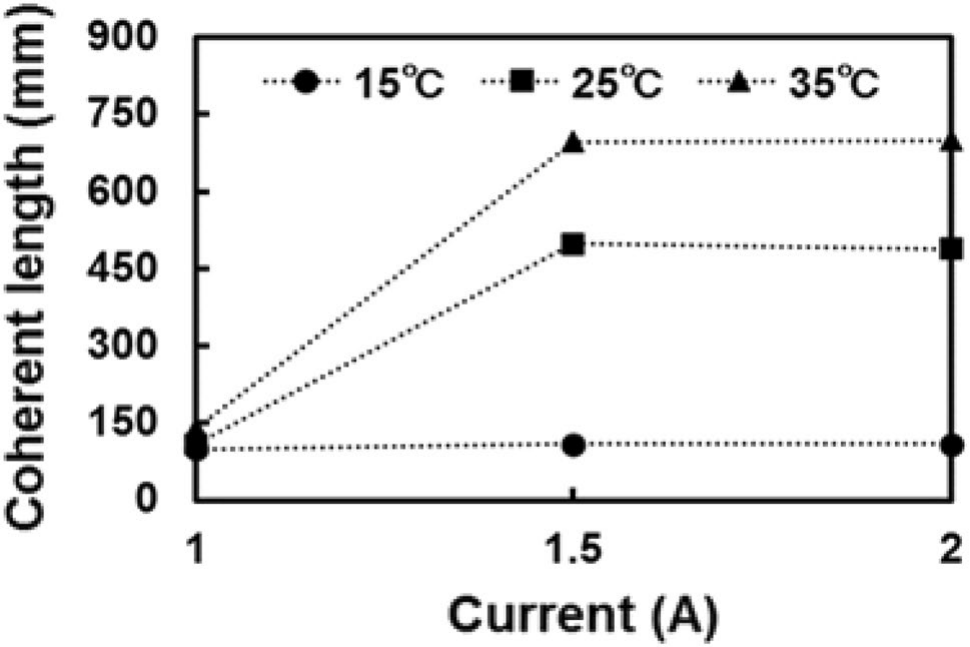
Coherence length relationship between current and temperature in the DFB-LD. The round, square, and triangle points represent the element temperature of 15 °C, 25 °C, and 35 °C, respectively. Coherence length can be controlled by adjusting the current and the temperature.

**Figure 3:**
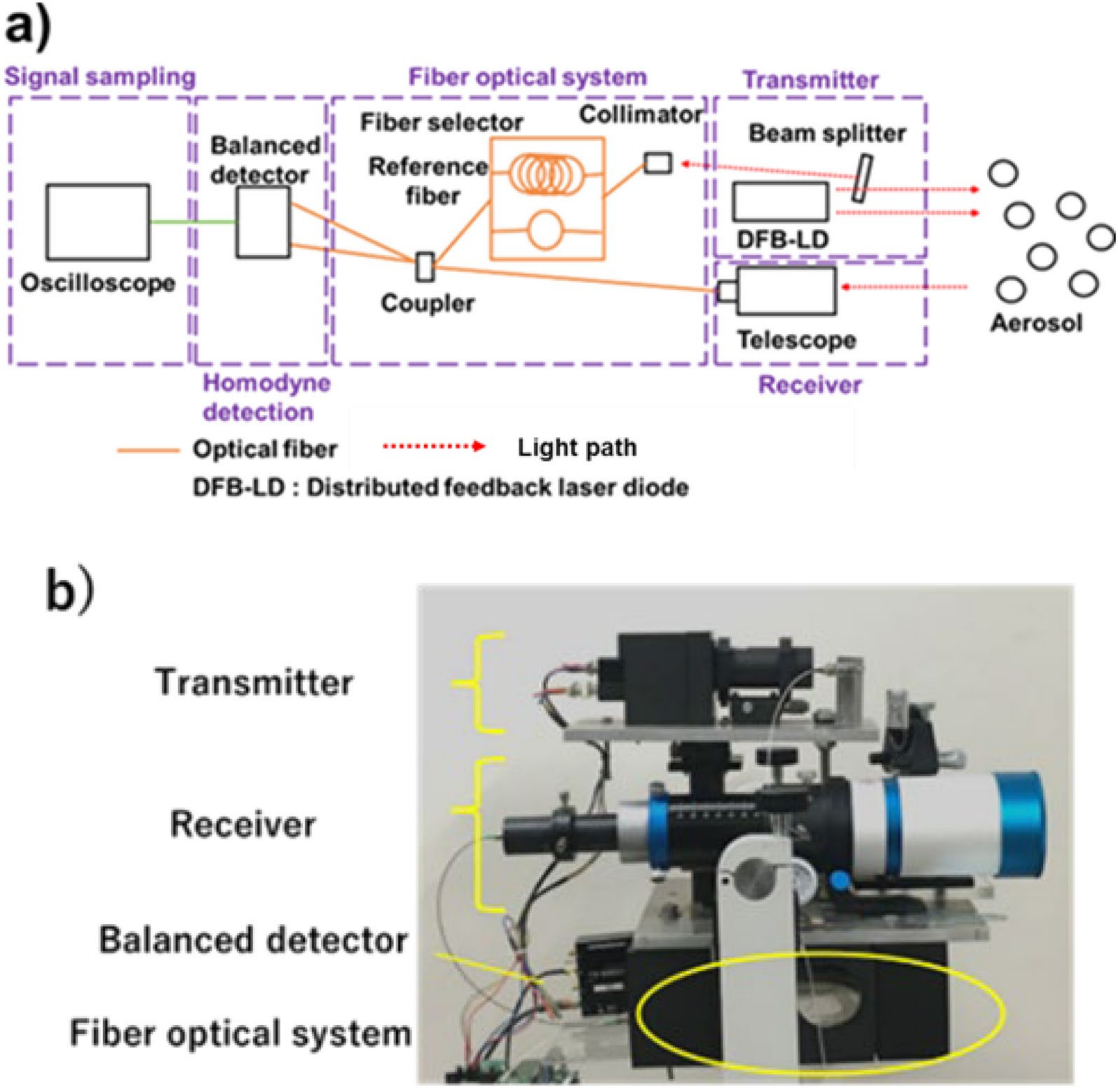
Description of LCDL system. (**a**) Schematic diagram of the LCDL system and (**b**) LCDL configuration. LCDL consists of transmitting, receiving, fiber optical system, homodyne detection, and signal processing.

The LCDL uses a 975 nm distributed feedback laser diode (DFB-LD) bulk element (Hamamatsu Photonics LE0697CWLD) as a light source. The output power of the DFB-LD is about 1 W. This output power enables the detection of dust under low concentrations. As a characteristic of the light source, a spectral width of a few picometers is necessary to achieve a high spatial resolution of 1 m or less. General DFB-LD has high wavelength stability and narrow spectral widths. They also have a small output power of a few 100 mW^39,40^. On the other hand, the spectral width of the DFB-LD used in this system can be changed^41^. The coherence length and the output power are adjusted by controlling the drive current and the element temperature. The relationship between the coherent length and the LD drive current is shown in Fig. 2. The coherence length of 0.8 m is accomplished when the drive current and element temperature are set to 2 A and 35 °C, respectively. Under this condition, the output power is 0.7 W. The LCDL system consists of a transmitter, a receiver, a fiber optical system, a homodyne detection, and a signal processing unit as shown in Fig. 3. For the transmitting system, a part of the light from the DFB-LD is introduced into the reference fiber by a beam splitter. This reference light intensity is optimized to around 1 mW. For the receiving system, a refracting telescope with an aperture of 61 mm is used. In the fiber optics, homodyne detection is conducted with the interference between the reference light and the received light on the 50:50 fiber coupler. Measurement areas can be arbitrarily determined by adjusting the reference fiber length using a fiber selector. FFT analysis is performed in the signal processing system. The sampling rate and acquisition time are set to 250 MSa/s and 5 ms, respectively. Each FFT frame is 10 μs and 500 frames are averaged to produce a high-resolution Doppler shift signal. The bandwidth of the balanced detector (BD) is 200 MHz. In FFT analysis, frequency resolution is inversely proportional to the time. The frequency resolution by FFT analysis is 0.1 MHz. Table 1 summarizes the parameters of the LCDL lidar.

**Table 1:** Specifications of LCDL.

Component	Parameters	Specification
Transmitter	Center wavelength	975 nm
	Laser power	0.7 W
	Coherence length	0.8 m
	Drive curent	2 A
	Element temperature	35 °C
Receiver	Diameter of aperture	61 mm
	Coupling ratio of 2 × 2 SM fiber coupler	50 :50 (λ = 980 nm)
	Light receiving sensitivity of balanced detector (PD)	0.75 A/W @ λ = 1.0 μm
	Bandwidth of balanced detector	200 MHz
Data acquisition	Sampling rate	250 MSa/s
	Sampling time	5 ms

## Experiments and results

**Figure 4:**
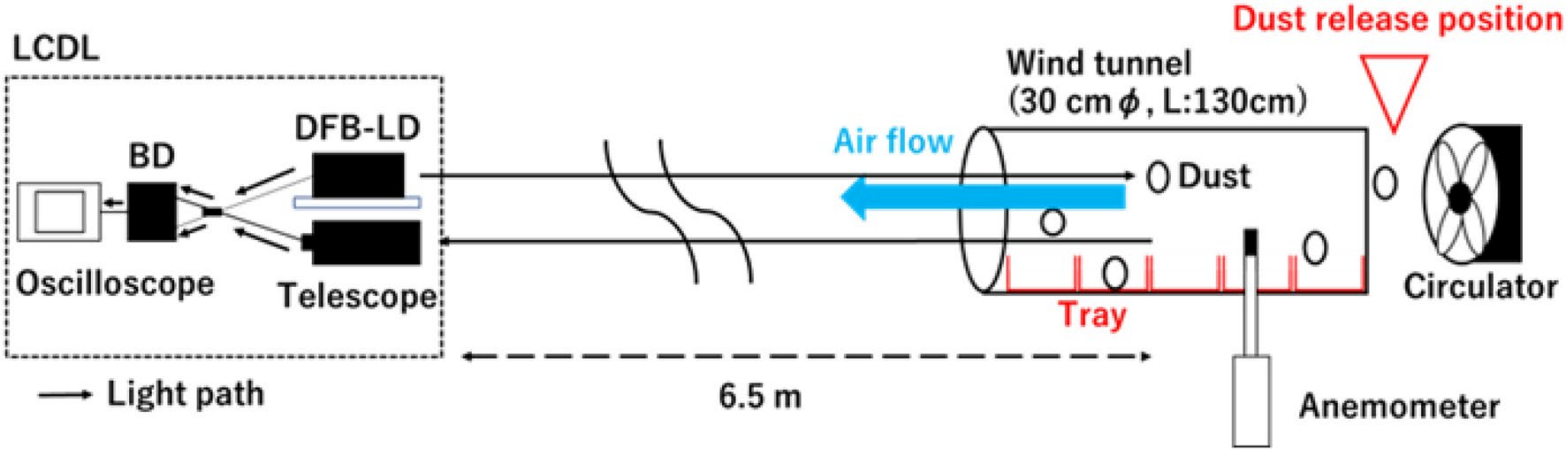
Dust flow experiment. The dust is released and flown through the wind tunnel by the wind. The dust flow is monitored by LCDL at 6.5 m from the wind tunnel, and the wind speed in the wind tunnel is simultaneously measured by an anemometer. The released dust is collected in the five trays placed inside the wind tunnel.

An indoor Doppler shift measurement is conducted using a wind tunnel box of length 130 cm and a square side of 30 cm using a circulator to suspend the dust. The schematic diagram of the experimental system is shown in Fig. 4. The scatterers used in the experiment are flour and calcium carbonate particles. The wind tunnel is placed approximately 6.5 m away from the LCDL. The wind direction from the circulator is a headwind in the direction of the lidar's line of sight. An anemometer (testo 405i Smart Probe) is placed inside the wind tunnel. The data from the small anemometer are then compared to the speed of dust particles measured by the LCDL. The dust is released from a position in front of the circulator, and the dust is scattered into the wind tunnel by the wind. Five trays are arranged at an interval of 25 cm inside the wind tunnel to collect the dust particles and to examine the particle size distribution of dust. The flour and calcium carbonate amounts released in 1 min are about 200 g and 130 g, respectively.

**Figure 5:**
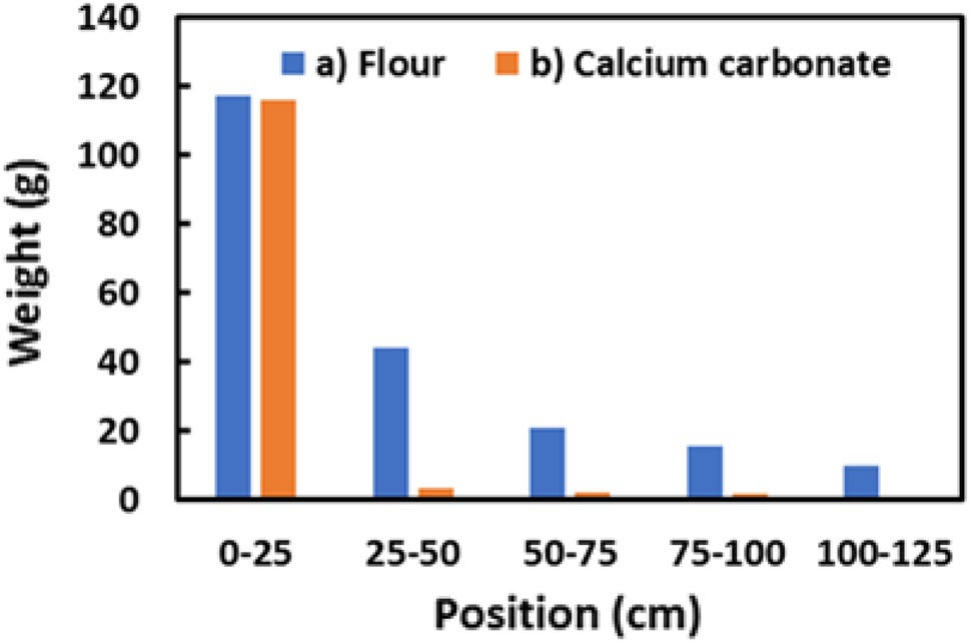
Relationship between weight of dust collected and distance of each tray position for (**a**) flour and (**b**) calcium carbonate particles. Most of the calcium carbonate particles are collected near 0 cm of the release position, while significant flour particles are collected up to 125 cm away from the release position.

Figure 5 shows the distribution of the dust weights collected on the trays inside the wind tunnel at 25 cm intervals. The zero position is the right under the position at which the dust was released. The results show that calcium carbonate particles are concentrated near the zero position, while the flour particles are widely distributed. These results are expected since the density of flour is smaller than that of calcium carbonate, indicating that flour particles flow in the air for a more extended time.

**Figure 6:**
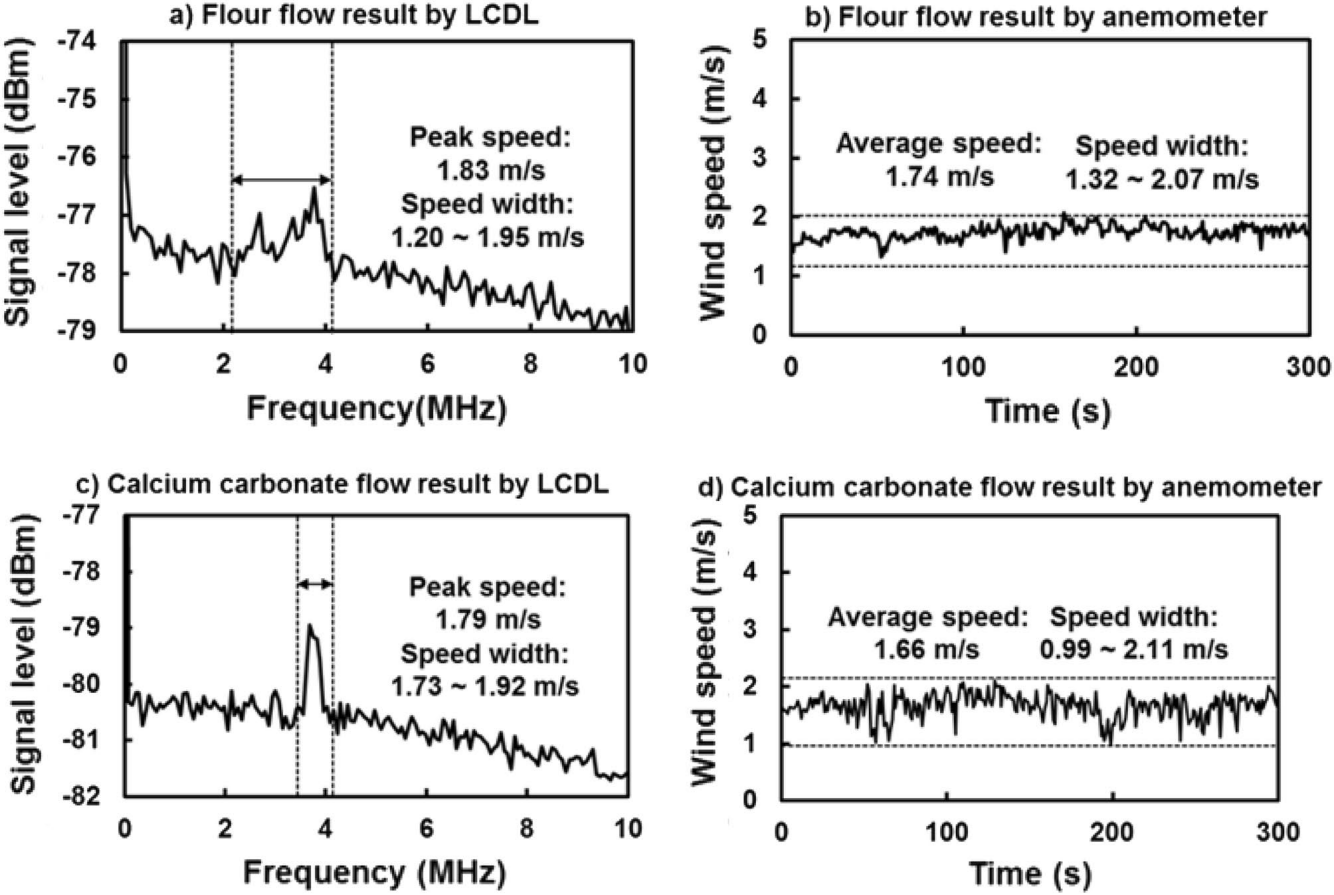
Dust flow signals of (**a**) flour particles from LCDL, (**b**) flour flow from the anemometer, (**c**) calcium carbonate particles from LCDL and (**d**) calcium carbonate flow from the anemometer. The LCDL results of calcium carbonate particles flow shows a narrower speed range compared to that of flour particles.

Figures 6a and b show the simultaneous FFT signals of LCDL and the wind speed of the anemometer when the flour was released. Figures 6c and d also show the results when calcium carbonate was released. In Figs. 6a and c, the acquired waveforms are the averaged result of 500 waveforms in 5 ms after their FFT analysis. In Figs. 6b and d, the time resolution of anemometer is 1 s. The Doppler shift frequency of the flour particle in Fig. 6a is 2.46–4.01 MHz with a peak position of 3.76 MHz, and these are equal to a speed width of 1.20–1.95 m/s with a peak speed at 1.83 m/s for particle flow. The anemometer results in Fig. 6b show a speed ranging from 1.32 to 2.07 m/s and a 5-min average speed of 1.74 m/s for air flow. This shows a good agreement between LCDL and anemometer results. Similarly, the Doppler shift frequency of calcium carbonate particles ranges from 3.54 to 3.95 MHz with a peak position at 3.68 MHz. These frequency shifts are equivalent to speed width from 1.73 to 1.92 m/s and a peak speed of 1.79 m/s. The anemometer results in Fig. 6d show the speed width from 0.99 to 2.11 m/s and these 5-min average speed of 1.66 m/s. Unlike flour particles result, the smaller range of frequency shifts for calcium carbonate means a narrower speed width. The spatial distribution of the flour particles in this wind field indicates that these particles tend to float in the air for a longer time. In contrast, calcium carbonate particles do not float in the air as much as flour particles, so their speed range is narrow. The anemometer waveforms of flour and calcium carbonate are also different. The anemometer waveforms for calcium carbonate particles have a spike due to the particles that fell rapidly in a short time. On the other hand, such a spike is not observed for flour particles and shows relatively slow fluctuations.

**Figure 7:**
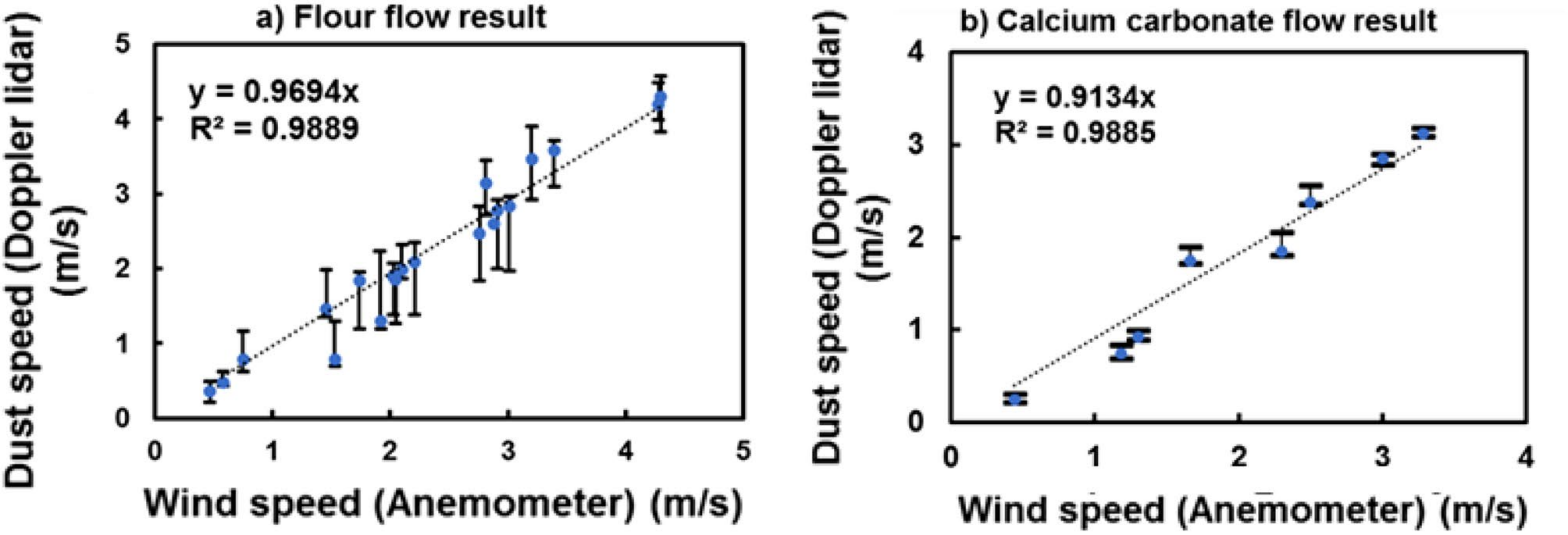
Relationship between dust speed of (**a**) flour particles and (**b**) calcium carbonate particles measured by LCDL and wind speed by the anemometer. A linear agreement is observed in the range of 0–5 m/s wind speed. The slope of calcium carbonate flow is lower than that of flour.

Figures 7a and b show the correlation between LCDL and anemometer speed measurements for the flows of flour and calcium carbonate particles, respectively. The blue markers represent the peak positions. The vertical lines attached to the markers represent the speed widths estimated from Doppler shift frequency widths. In the speed range from 0 to 5 m/s, the measured speeds from LCDL and anemometer show a good linear agreement. In the case of flour particles, the peak positions are not centered in their speed width. Overall, the speed width of flour is about 0.7–1 m/s, while that of calcium carbonate is narrower at about 0.3 m/s. The slope of calcium carbonate flow is lower than that of flour. This difference shows the inherent nature of the dust particles and is discussed in the next section.

## Discussion

The measurement results for flour and calcium carbonate particles showed differences in speed width (Doppler shift frequency width). In the experiment, the dust particles are collected at every fallen position. Their weight distribution reflects the particle radius and amounts. We simulate the dust flow in terms of the particle’s mass and particle radius. If there is no horizontal wind, the dust will fall directly after release. However, the horizontal position from which the dust particles fall shifts under the influence of crosswinds. In the experiment, the wind flows to the direction of the lidar's line of sight with a speed of 3.5 m/s. The dust falls to ground within the crosswind of 3.5 m/s. The experiments were conducted in the wind speed range 0–5 m/s, and simulations are conducted at 3.5 m/s to match the experimental results. Under the experimental setup, the vertical distance traversed by a dust particle is assumed to be 30 cm, and viscous air resistance is assumed. The particle speed in a viscous resistance is given by Eq. (2):

$${V}_{x}\left(t\right)= {v}_{0}\left(1-exp\left(\frac{-k}{m}t\right)\right)$$

2 $${V}_{y}\left(t\right)= \frac{mg}{k}\left(1-exp\left(\frac{-k}{m}t\right)\right)$$

where *m* is the mass of a dust particle; *g* is gravitational acceleration; *k* is the coefficient of air resistance; and *t* is the time of travel. The coefficient of air resistance is assumed to follow Stoke’s law (6πμr). Equation (2) is integrated and transformed into an equation for the falling distance. The mass of the dust is calculated from the time and distance over which the dust falls. Since this dust mass reflects the density and volume, the radius of the dust can be estimated. The densities of flour and calcium carbonate are 600 kg m^−3^ and 2600 kg m^−3^, respectively. The particle radius is converted from the mass to obtain the extinction coefficient *α* by Eq. (3):

3 $$\alpha =N\pi {r}^{2}{Q}_{ext}$$

where *N* is the number of particles per volume, *r* is the particle radius, and *Q*_*ext*_ is the extinction efficiency. As flour and calcium carbonate particles are sufficiently larger than the optical wavelength, *Q*_*ext*_ can be assumed to be equal to 2.

**Figure 8:**
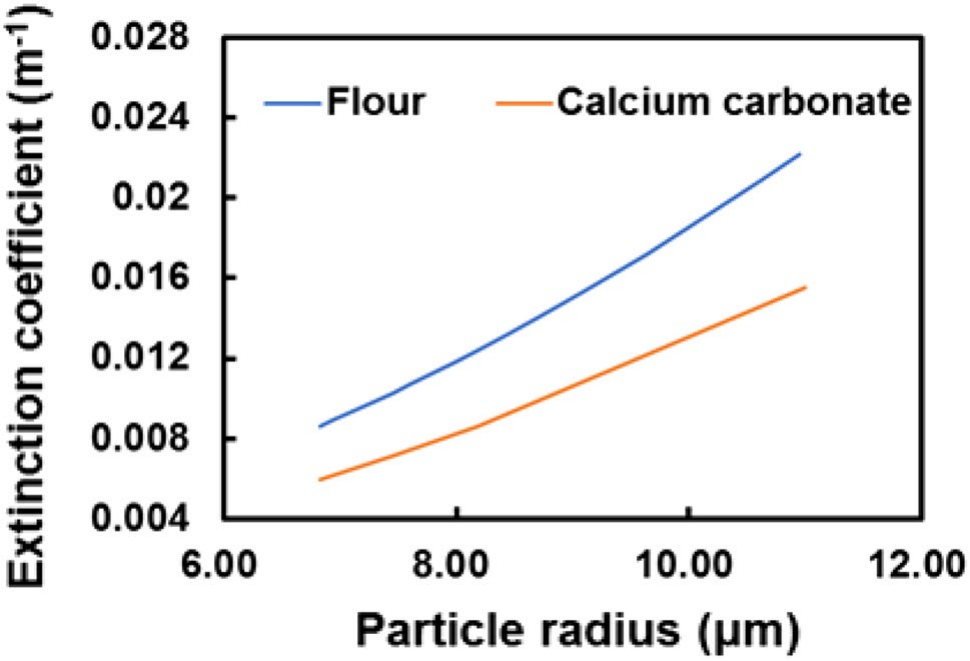
Extinction coefficient simulation for flour and calcium carbonate. The flour density is set to 600 kg m^−3^ and calcium carbonate density is 2600 kg m^−3^. The extinction coefficient of flour is larger than that of calcium carbonate because the number of flour particles released is larger than that of calcium carbonate.

**Figure 9:**
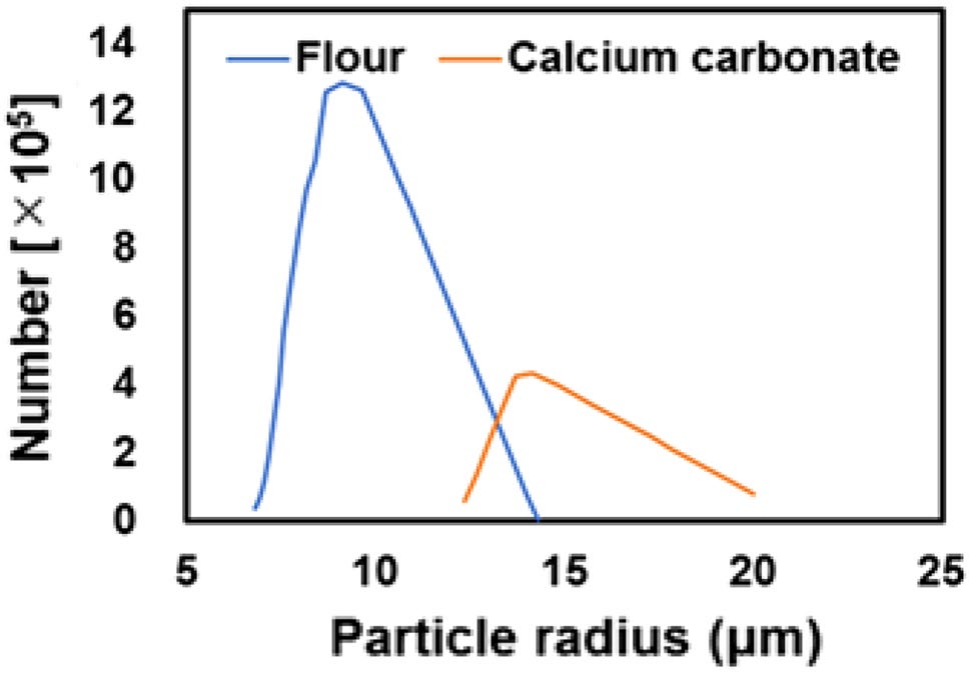
Particle size distribution of flour and calcium carbonate. The vertical axis represents the number of particles, and the horizontal axis represents the particle radius.

Based on the extinction coefficient, the interference signal of LCDL is simulated. The calculated results of the extinction coefficients are shown in Fig. 8. The particle extinction coefficient of flour is larger than that of calcium carbonate. This result can be attributed to the number of particles N based on this experiment. Flour particles had a smaller average particle radius and higher particle counts than calcium carbonate. Figure 9 shows the calculated particle size distribution based on the experimental results of the weight distribution of dust in Fig. 5. The interference distance of LCDL is 80 cm, and the magnitude of the interference signal varies depending on the distance. The interference signal distribution is assumed to be a Gaussian distribution. After converting from mass to number of particles, the particle size distribution is obtained by considering the interference signal distribution. The mode radius of flour and calcium carbonate particles are estimated to be around 9 μm and 14 μm, respectively. Since the fallen position of particles reflects particle radius, density, and wind speed, the relationship between Doppler frequency (speed) and number of particles can be discussed.

**Figure 10:**
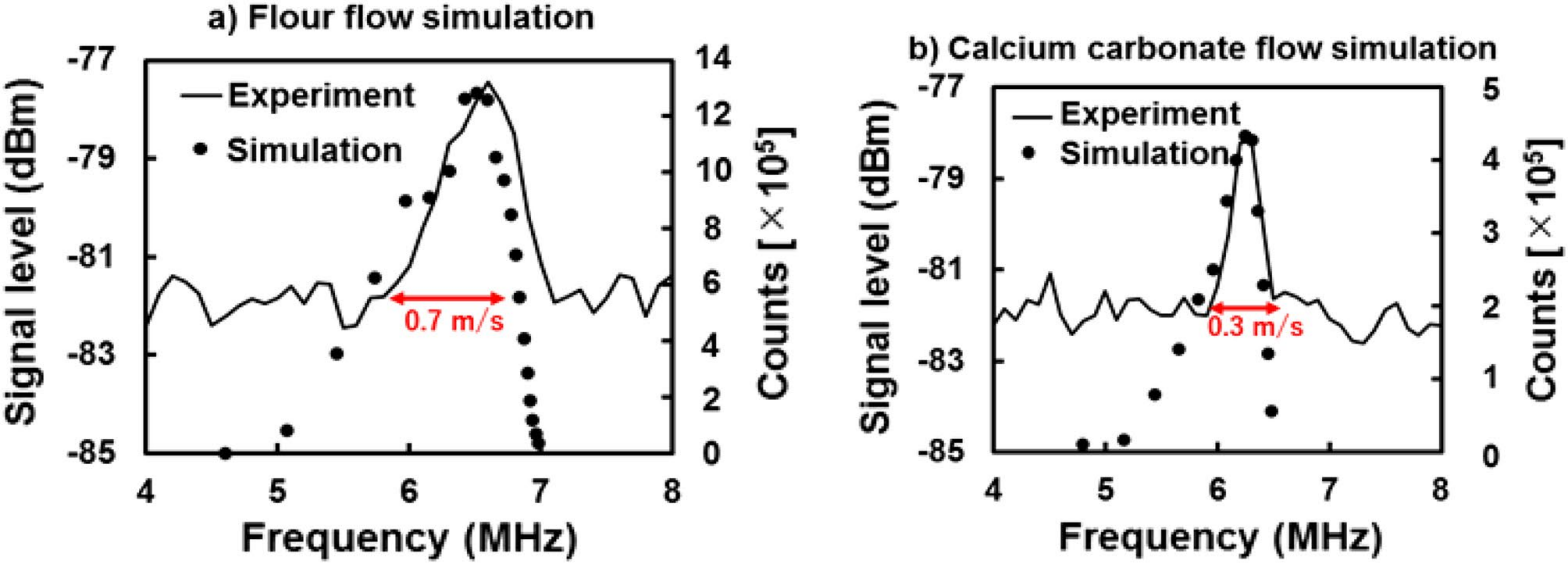
Doppler signal simulations of (**a**) flour and (**b**) calcium carbonate particle flow. The lefthand side vertical axis of the LCDL experiment result indicates the signal level (dBm), and the righthand vertical axis of the simulation shows the number of particles (counts). The horizontal axis is the frequency. The wind speed is simulated at 3.5 m/s (= 7 MHz).

Lidar signal simulation with the experimental results of dust weight distribution is performed, too. Figure 10 shows simulated results (black circle) of (a) flour and (b) calcium carbonate particle flow together with LCDL experimental results (solid line). The simulation results have shown to coincide well with LCDL signals. Simulated signal distributions are reflected by the particle size distribution in Fig. 9. The flour particles have a wider full-width speed range of 0.70 m/s compared to calcium carbonate having a narrower full width speed range of 0.3 m/s. The shift of the Doppler frequency peak from the center to the higher frequency side contributes to the fact that the mode radius of the particles is biased on the smaller side than the center with respect to the particle size distribution, as shown in Fig. 9. The Doppler shift frequency of flour particles reaches up to 7 MHz, whereas that of calcium carbonate particles is only about 6.5 MHz, indicating that the flour flow has a high-speed component. In addition, the particle flow on the high-frequency side is sharp, while the rise on the low-frequency side is gradual, indicating that the particles with slower velocities greatly widen the speed range. As a result, this confirms that particle size distribution can be estimated by LCDL Doppler signal. In this simulation, when the wind speed is set at 3.5 m/s, this produces a difference of 0.2 MHz between peak positions of flour and calcium carbonate flow. The difference between peak speeds represents the difference in dust speed due to the mode radius. Figure 7 also shows that the slope of calcium carbonate particles is lower than that of flour. This indicates that the net force on the calcium carbonate particles in the direction of the fall is more significant due to the heavier mass of calcium carbonate particles, resulting in a lower speed in the line-of-sight direction of the LCDL. The high-frequency resolution of the LCDL system allows us to capture these two slight differences in flow in the order of a few centimeters and slight differences in particle radius in the order of a few micrometers. The analysis of the LCDL signals from flour and calcium carbonate flow measurements can provide information on the size distribution of suspended dust based on the observed dust speed. Simulations have been performed under several wind speeds from 0 to 5 m/s and the simulated Doppler shift frequency width is equivalent to the experimental results. LCDL can identify dust and help monitor its spatial distribution. The signal-to-noise ratios of simulated signals are large (~ 7 dB), while the experimental system has a signal-to-noise ratio of 3 dB. The system's low signal-to-noise ratio comes from the lower coupling ratio between the transmitting and receiving optics. This result indicates that the efficiency of the lidar system can be improved to have a higher signal-to-noise ratio which leads to the detection of the wind itself, i.e., the atmosphere flow.

## Conclusion

This work shows that the LCDL system can measure near-ground dust flow with high temporal and spatial resolutions. The dust speed detected by the LCDL system shows a good relationship with measurements from the anemometer. LCDL can also monitor different dust flows. Simulations under the experimental condition show that differences in speed width are due to dust particle radius and mass. Based on the speed distribution obtained by LCDL, the particle size distribution can be inferred, and the differences in size distribution can provide information on the type of suspended dust in the atmosphere. Furthermore, this work has shown that LCDL can aid in the detailed visualization of dust flow in real-time. This result allows for further discussion and interpretation of the mixing and interaction between surface-derived dust and the lower atmosphere. This is the next step that we will explore to have a comprehensive and detailed dataset that captures the movement of dust in actual fieldwork.

## Data Availability

Data will be made available from the corresponding author on request.
